# *Anopheles *species associations in Southeast Asia: indicator species and environmental influences

**DOI:** 10.1186/1756-3305-6-136

**Published:** 2013-05-04

**Authors:** Valérie Obsomer, Marc Dufrene, Pierre Defourny, Marc Coosemans

**Affiliations:** 1 Department of Parasitology, Prince Leopold Institute of Tropical Medicine, Nationalestraat 155, Antwerp 2000, Belgium; 2 Earth and Life Institute, Environmental Sciences, Université Catholique de Louvain, Croix du Sud 2/16, Louvain-la-Neuve B-1348, Belgium; 3 Biodiversity and landscape department, Gembloux Agro-BioTech, University of Liège, passage des déportés, 2, Gembloux, B-5030, Belgium

## Abstract

**Background:**

Southeast Asia presents a high diversity of *Anopheles*. Environmental requirements differ for each species and should be clarified because of their influence on malaria transmission potential. Monitoring projects collect vast quantities of entomological data over the whole region and could bring valuable information to malaria control staff but collections are not always standardized and are thus difficult to analyze. In this context studying species associations and their relation to the environment offer some opportunities as they are less subject to sampling error than individual species.

**Methods:**

Using asymmetrical similarity coefficients, indirect clustering and the search of indicator species, this paper identified species associations. Environmental influences were then analysed through canonical and discriminant analysis using climatic and topographic data, land cover in a 3 km buffer around villages and vegetation indices.

**Results:**

Six groups of sites characterized the structure of the species assemblage. Temperature, rainfall and vegetation factors all play a role. Four out of the six groups of sites based on species similarities could be discriminated using environmental information only.

**Conclusions:**

Vegetation indices derived from satellite imagery proved very valuable with one variable explaining more variance of the species dataset than any other variable. The analysis could be improved by integrating seasonality in the sampling and collecting at least 4 consecutive days.

## Background

Southeast Asia presents a high diversity of *Anopheles* including more than 30 *Anopheles* species present in the domestic environment [1]. The major vectors, *Anopheles dirus sensu lato*, *An. minimus s.l*., *An. epiroticus*, are responsible for most malaria cases in the region but secondary vectors might play a sporadic role [2]. Environmental requirements differ for each species and should be clarified because of their influence on malaria transmission potential. Various research studies [3,4] try to relate *Anopheles* species and environmental drivers in the region but are often restricted to small areas and a few species due to the logistic effort necessary to obtain optimal sampling. On the other hand, monitoring projects can generate vast quantities of data on a wider scale, but sampling design is often not optimal for exploring biodiversity issues. This is the case of the MALVECASIA project [5], which operated a major collection effort throughout Laos, Cambodia and Vietnam capturing *Anopheles* between 2003 and 2005 in more than a hundred sites to monitor insecticide resistance. For logistic reasons, surveys were not concurrent. In this context studying species associations and their relation to the environment offer some opportunities. Associations are less subject to sampling error than individual species. Moreover, some species such as *An. dirus s.l.* are difficult to collect and abundance varies greatly according to the rain history of the previous days [6]. Occurrence of such elusive species could be revealed by the presence of associated species.

Several concept of species association have been developed [7] but Fager and McGowan [8] simply refer to a recurrent group of co-occurring species. Cole [9] designed a first coefficient measuring the degree of association between pairs of species which was used to identify association between larvae [10-12]. The index was further corrected by Hurlbert [13] to account for species frequencies bias and used for analysis of mosquito association [14-19]. Other indices target species dominance [20-22]. Southwood [23] also developed an index that takes the number of individuals collected into consideration [24]. However, only pairs of mosquito species were investigated and mostly using a count of common breeding sites at larval stages [25].

Studying associations between more than two species and particularly *Anopheles* species adults associations can be a challenge: (1) abundance data may not reflect the true proportion of species because some species are more easily captured than others, (2) correlation coefficients can’t be used as they associate co-occurring species only if their abundances vary linearly, (3) double absences should be discarded as they do not mean association, and (4) false absence are common in particular when studying a great number of species with different seasonality, behavior and response to sampling [26]. Nevertheless, this paper proposes a method to tackle each of the above mentioned issues and apply an ecological concept based on indicative species to identify species association.

In this context, this paper aims to (1) define species assemblages and identify indicator species for those assemblages, and (2) search environmental determinants which could explain or help delineate those assemblages.

## Methods

### Entomological data

**Figure 1:**
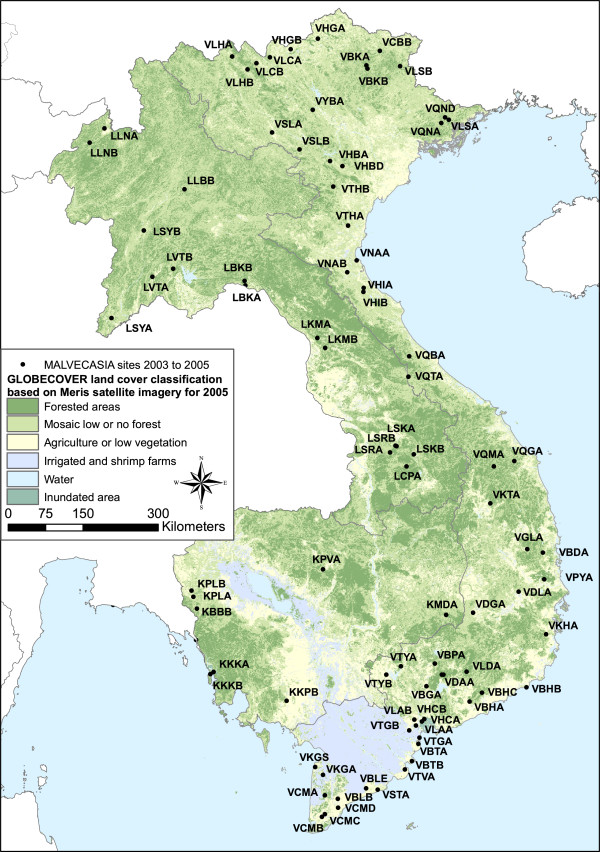
**Map of the survey sites. **Each site has a 4 digit codes corresponding to description in Van Bortel *et al*. (2008). Background is based on Globcover (Defourny *et al*., 2009).

The MALVECASIA dataset described in Van Bortel [5] investigated approximately two sites per province in Laos, Cambodia and Vietnam from 2003 and 2005 (Figure 1) (can be obtained from the author M. Coosemans). The present study concentrates on adult *Anopheles* captured by human baits indoors and outdoors, which correspond to *Anopheles* of interest for public health. All sites were surveyed during two to twenty one nights just before or after the rainy season. Sites with less than 3 nights of collection were discarded as well as sites with no mosquito recorded. Species were defined on their morphological characteristics and sibling species of the *An. dirus*, *An. minimus* and *An. maculatus* complex were not considered separately. Similarly species members of the *Annularis* group were mixed together. *An. pampanai* is also present in the region but was misidentified to be *An. minimus* in a couple of sites and thus discarded. Species occurring in less than 4 sites were also discarded. This includes *An. argyropus*, *An. baileyi*, *An. crawfordi*, *An. indefinitus*, *An. lesteri*, *An. lindesayi*, *An. pseudojamesi* and *An. varuna* (Table 1). The abundance values were weighted per man nights and recorded using the transformation (log (Abundance+1)) [27,28] in order to give less weight to the few very abundant species.

**Table 1:** MALVECASIA entomological dataset

**Taxonomy**	**Code**	**Species**	**Total captured**	**Sites (No.)**
Genus *Anopheles*				
Subgenus *Anopheles*				
*Anopheles* series				
Myzorhynchus series				
Barbirostris group				
barbirostris	BARB	*An. barbirostris*	2014	21
campestris	CAMP	*An. campestris*	16	4
Hyrcanus group				
nimpe	NIMP	*An. nimpe*	1787	9
peditaeniatus	PEDI	*An. peditaeniatus*	5171	17
sinensis (karyotype)	SINE	*An. sinensis*	9324	44
Umbrosus group				
umbrosus	UMBR	*An. umbrosus*	164	3
Subgenus cellia				
Myzomyia serie				
Funestus group				
aconitus (karyotypes)	ACON	*An. aconitus*	10085	38
jeyporiensis (karyotypes)	JEYP	*An. jeyporiensis*	7090	24
minimus (complex)	MINI	*An. minimus*	24993	32
Neocellia serie				
Annularis group	ANNU	*Annularis group*	15985	37
annularis				
nivipes (complex)				
pallidus				
philippinensis				
Jamesii group				
jamesii (karyotypes)	JAME	*An. jamesii*	2737	11
splendidus	SPLE	*An. splendidus*	1376	25
Maculatus group				
maculatus	MACU	*An. maculatus*	11459	52
No group				
karwari (karyotypes)	KARW	*An. karwari*	1263	7
Neomyzomyia serie				
Kochi group				
kochi	KOCH	*An. kochi*	2749	10
Leucosphyrus group				
dirus (complex)	DIRU	*An. dirus*	8705	29
Tessellatus group				
tessellatus	TESS	*An. tessellatus*	1543	28
Pyretophorus serie				
No group				
subpictus (complex)	SUBP	*An. subpictus*	3068	6
epiroticus (complex)	EPIR	*An. epiroticus*	32047	21
vagus (karyotypes)	VAGU	*An. vagus*	18714	20
	**Total**		**160290**	**86**

Taxonomic level and mosquito collection information.

### Environmental data

Four groups of variables were investigated: (1) XY: Spatial geographical coordinates of latitude and longitude and their second polynomial combinations to evaluate the significance of spatial autocorrelation, (2) CT: abiotic factors such as climatic trends and topography from the Worldclim dataset [29] and the CRU CL2.0 dataset [30] as well as elevation, slope, flow direction, flow accumulation and compound topographic index available from the USGS digital elevation model, (3) GC: variables derived from land cover GLOBCOVER at a resolution of 300 m derived from Meris satellite annual composite image for year 2005 and which provides harmonized classes over the three countries [31], (4) ND: vegetation indices including the annual greenness of vegetation (NDVI) as well as wetness index for vegetation (NDWI) derived from spot VEGETATION satellite yearly composite images for 2005 at 1 km resolution. NDVI and NDWI layers were calculated using software ENVI 4.4 and are based on annual composites of daily spot VEGETATION images based on the mean compositing method [32]. The environmental values were extracted at each site. The minimum, maximum, mean and standard deviation value over a buffer area of 3 km around each site was extracted for vegetation indices (ND) using Arcgis 9.3. The proportion of each land cover classes was also estimated in a buffer or 3 km around each site. Landscape indices including fragmentation were extracted using the software FRAGSTATS [4,33]. The GLOBCOVER dataset has some well known misidentification of forest zones in the south of Vietnam. The layer was corrected using a mask based on NDVI (<0.5) and NDWI (<0.3) value of spot vegetation annual composite for year 2005.

### Analytical strategy

**Figure 2:**
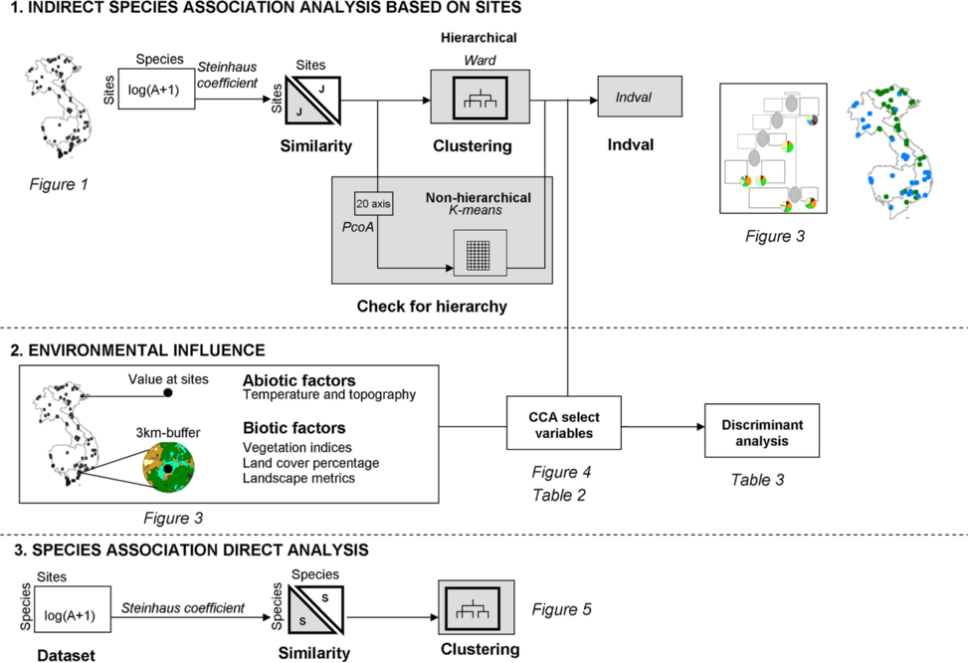
General scheme of analysis.

Species associations are analyzed using indirect clustering of species through three major steps: calculation of similarities between pair of sites according to species, direct clustering of the sites based on those similarities, then analysis of the Indicator Value for each species at each clustering level. Environmental influences are then investigated. The general scheme of the analysis is presented in Figure 2.

Similarities between pair of sites according to species are calculated in R software [34] based on *Anopheles* abundance aggregated per sites using Steinhaus asymmetric coefficient [35]. Those similarities are then used for hierarchical clustering of sites with Ward’s minimum variance method [36]. To confirm presence of hierarchical structure in the data, we followed suggestion of Dufrêne and Legendre [28] by using the k-means method [37] on the sites coordinates on the 20 first axis of a Principal Coordinates Analysis ordination (PCoA) [38] based on the similarity matrix.

Once sites are classified in clusters, indicator species corresponding to the various clusters of the site typology are identified using the IndVal method [28] (Additional file 1). The most representative species is identified for each cluster of sites and at each level of the cluster tree. The indicator value is calculated independently for each species, thus dealing with differential response to sampling. Indicator species are defined as the most characteristic species of each group, found mostly in a single group and present in the majority of the sites belonging to that group. This index is maximum (= 100%) when all specimens of a species are found in a single group of samples and when the species occurs in all samples of that group. The basic idea is to measure the species indicator value for all the levels of a hierarchical typology. The IndVal index allows also identifying species typical for the intermediate level of the clustering history. The statistical significance of the species indicator values is evaluated using a randomization procedure [28]. Associated species are species which are indicators of the same cluster of sites.

### Environmental influences

#### Select useful environmental data

Canonical Correspondence Analysis (CCA) available in the software canoco 4.5 for windows [39] quantifies and describes the relationship of a particular set of variables with species assemblages [40,41]. CCA has the advantage of being less influenced by noise in species abundance and by inter-correlated environmental variables than other methods. Relevant variables were then selected using a Monte-Carlo randomization test with 499 steps in an initial CCA with all variables and the ones that proved not to be active (p>0.05) were removed from the analysis [40].

#### Canonical analysis per groups of environmental variables and variance partitioning

The four groups of variables are analyzed separately to perform variance partitioning [40] and identify which group of factors has an overall influence on the distribution. For each group of variables XY, CT, GC, ND the variables were integrated in a stepwise manner into a canonical analysis. The process was continued till a maximum of 5 variables were integrated and using only significant variables (Monte Carlo test). The best performing variables were plotted against the species sample in order to analyze the influence of variables on the occurrence of the species.

#### Environmental justification to clustering of sites based on species

The best performing environmental variables are used to perform a multivariate discriminant canonical analysis in software SAS 8.2. This process analyzes which groups can be differentiated by linear combinations of environmental variables. The process identifies the best explanatory variables and a discriminant analysis using those variables gives an idea of the rate of omission and commission errors if sites classification were only based on specific linear combination of environmental variables.

## Results

### Indirect species assemblage

**Figure 3:**
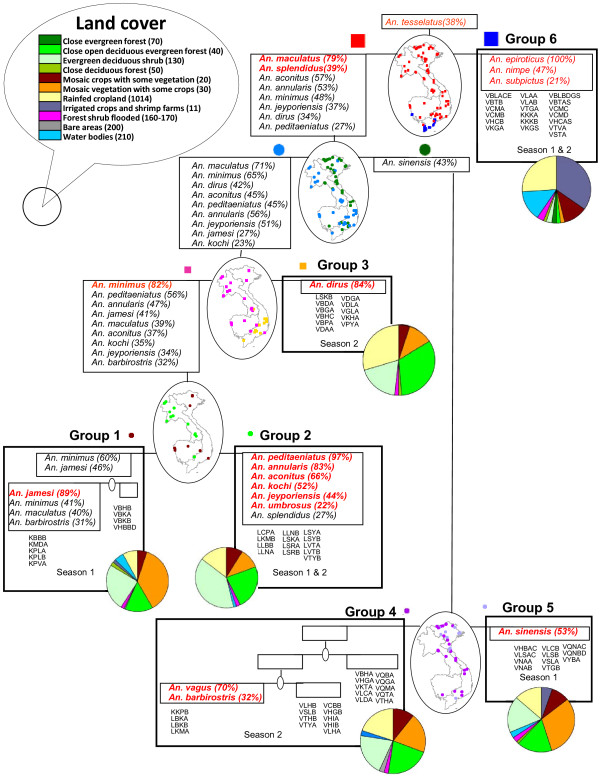
**Indirect cluster and indicator species. **Indirect clustering and indicator species for 6 main groups based on sites similarities in terms of abundance of species. For each group, species showing a significant association (only *An. tesselatus *in node 1 is not significant) characterized by an indicator value >20% (in brackets) are listed. The species are displayed in red font when they present the highest indicator value obtained by that particular species during the analysis. A small map is presented at each node showing distribution of the two separating clusters. A pie presents the proportion of the various land cover calculated as the mean over the sites of the group. The sites included in the group are listed under the groups (starting with V: Vietnam, C: Cambodia, L: Laos) as well as the season of collection.

Figure 3 presents the clustering of sites using the Ward method and subsequent indirect clustering of species. The cluster of sites is first built based on site similarities in terms of abundance of species. At each level of separation between groups, the indicator value is calculated for each species. Species presenting an indicator value significant and higher than 20% are associated to the group of sites (Figure 3). A small map is presented at each node of a cluster to see spatial distribution of the two separating clusters. A pie presents the proportion of the various land cover calculated as the mean divided by the sites of the group.

Six groups of sites can be defined with high indicator value for at least one species. The most ubiquitous species seems to be *An. tesselatus*. This species is indicative of the root node being associated with no site in particular, but the result is non significant. The most different group of sites is Group 6 (first node) with 19 sites characterized by brackish water including mangrove or shrimp farms in South Vietnam and Cambodia. *An. epiroticus* is very indicative of this group (100%) as to a lesser extend *An. nimpe* (47%) and *An. subpictus* (21%). Those three species are thus associated. *An. subpictus* was mostly found in the sites investigated in the first part of the year (season 1).

*An. maculatus* and *An. splendidus* are indicator species for the sites not included in Group 6. These species are ubiquitous and can thus be found if all subsequent groups. It is however important to keep in mind that *An. maculatus* is a complex of species and this ubiquity might be linked to the occurrence of several sibling species with different environmental requirements. This group of sites further divides into one group with no specific species and another group represented by *An. sinensis*. The *An. sinensis* branch separates in Group 5 characterised by *An. sinensis* and mostly found in Vietnam, and Group 4 with many sites but only four sites which further associate with *An. vagus* and *An. barbirostris.* The land cover class post flooding or irrigated cropland which includes shrimp farms is clearly present in Group 6 of *An. epiroticus* but can be found in Group 5 of *An. sinensis*.

The rest of the sites which separated at node 2 provide Group 3 with only *An. dirus* as indicator species, suggesting no species association. *An. dirus* is also an indicator for higher hierarchical level (node 2) but reach its maximum for the group 3, which seems to be thus the typical type of sites for the species, at least in the second part of the year (season 2). A larger proportion of closed evergreen and closed to open deciduous evergreen forest characterize these sites located in Central Vietnam. *An. minimus* presents the highest indicator value for the rest of the sites. Those sites further separate in Group 1 characterized by the presence of *An. jamesi* and Group 2 where a large group of species are indicators. Those species include *An. peditaeniatus*, *An. annularis*, *An. aconitus*, *An. kochi*, *An. jeyporiensis* and *An. umbrosus*. No particular land cover could be associated with those species.

### Environmental influence

#### Species and environmental variables

**Figure 4:**
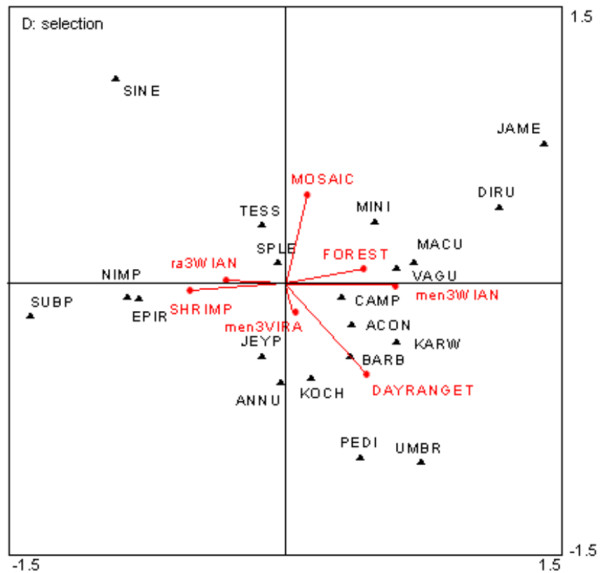
**Canonical analysis. **Bi plot based on canonical analysis for selected environmental variables. Mosquito species are presented in upper cases code of 4 letters with ACON: *An. aconitus*, ANNU: *An. annularis*, BARB: *An. barbirostris*, CAMP: *An. campestris*, DIRU: *An. dirus s.l.*, EPIR: *An. epiroticus*, JAME*: An. jamesi*, JEYP: *An. jeyporiensis*, KARW: *An. karwari*, KOCH: *An. kochi*, MACU: *An. maculatus*, MINI: *An. minimus s.l*., PEDI: *An. peditaeniatus*, SPLE: *An. splendidus*, TESS:*An. tesselatus*, SINE: *An. sinensis*, SUBP: *An. subpictus*, UMBR: *An. umbrosus*, VAGU: *An. vagus*. Environmental variables are surrounded by rectangles and abbreviations are as follow: MOSAIC: Mosaic vegetation and crop, FOREST: Dense forest, ra3WIAN: Range of variation of wetness index in 3-km buffer, SHRIMP: Shrimp farms, DAYRANGET: Temperature range, men3WIAN: Mean annual wetness index in 3-km buffer, men3VIRA: Yearly vegetation greenness variation (season).

Highly significant variables (Monte Carlo permutation test) explaining 5% or more of the variance are presented in Table 2. The ND greenness indices and GC land cover groups of variables perform well by explaining more than 45% of the variance each alone but with only 3 variables for the ND group against 5 variables for the GC group. The interaction between the two groups of variables is around 24%. The best performing variables were used to build a final graph (Figure 4). *An. epiroticus*, *An. nimpe* and *An. subpictus* seemed to be characterised by the presence of a shrimp farm (SHRIMP) and fragmented landscape (ra3WIAN). There is a clear opposition with *An. dirus* characterized by high dense forest (FOREST) and a high level of vegetation wetness index (men3WIAN). Mosaic vegetation and crop (MOSAIC) explain another dimension and seems more associated with *An. minimus* and *An. jamesi*.

**Table 2:** Environmental variables selected for the analysis and variance

**Variable description**	**Code**	**Contribution**
Spatial factor: spatial coordinates		
longitude * latitude	XY	13%**
**CT Abiotic factors: meteorology and topography**		
Precipitation of Driest Month	**MINRAIN**	5%**
Precipitation Seasonality (Coefficient of Variation)	**SEASONRAIN**	5%**
Lowest number of rainy days in a month	CMINRD0	7%**
Highest number of rainy days per month	CMAXRD0	10%**
Mean number of rainy days per month	CMEANRD0	5%**
Number of months with less 5 rainy days	CNBML5DAY	11%**
Mean Temperature of Driest Quarter	BIO_9	12%**
Precipitation of Warmest Quarter	**RAINWARMQ**	5%**
Number of months with mean temp<20°C	CNBMLESS20	6%**
Minimum temperature of the warmest month	**MAXMINT**	7%**
Maximum temperature of the coldest month	MINMAXT	9%**
Minimum temperature of the coldest month	MINMINT	14%**
Annual Mean Temperature	MEMET	10%**
Mean Diurnal Temperature Range	**DAYRANGET**	12%**
Temperature Annual Range (bio5-bio6)	BIO_7	14%**
Elevation above sea level (m)	ALT	10%**
Compound topographic index*100	CTI2	11%**
Slope*100	SLOPE3	6%**
**ND Biotic factors**		
Mean value in buffer 3 km for annual NDWI from 2003 to 2005	**men3WIAN**	18%**
Mean value in buffer 3 km for annual NDVI from 2003 to 2005	men3VIAN	16%**
Range of value in buffer 3 km for annual NDVI from 2003 to 2005	ra3VIAN	5%**
Range of value in buffer 3 km for annual NDWI from 2003 to 2005	**ra3WIAN**	5%**
Mean value in buffer 3 km for maximum NDVI from 2003 to 2005	men3VIMAX	14%**
Minimum value in buffer 3 km for maximum NDVI from 2003 to 2005	min3VIMAX	17%**
Mean value in buffer 3 km for range NDVI from 2003 to 2005	**men3VIRA**	9%**
Minimum value in buffer 3 km for annual NDVI from 2003 to 2005	min3VIAN	17%**
Minimum value in buffer 3 km for annual NDWI from 2003 to 2005	min3WIAN	18%**
Maximum value in buffer 3 km for annual NDVI from 2003 to 2005	max3VIAN	13%**
Maximum value in buffer 3 km for annual NDWI from 2003 to 2005	max3WIAN	16%**
**GC Land cover**		
1 Forest (40,50,60,70,80,100,110,30) percentage area 3 km buffer (PCA)	GFPCA1	10%**
1 Forested areas (40,50,60,70) (PCA)	**FOREST**	6%**
40 Closed/ open broadleaved/ evergreen/ deciduous forest (100) (PCA)	GDPCA40	6%**
30 Mosaic veg. (grassland/ shrub/ forest) (60%)/ cropland (35%) (PCA)	**MOSAIC**	5%**
130 Closed/ open (broadleaved/ evergreen/ deciduous) shrub (PCA)	**SHRUB**	8%**
5 Irrigated or shrimp farms (11) (PCA)	**SHRIMP**	12%**
No. of Patches (NUMP) 1 forest (40,50,60,70,80,100,110,30)	**PATCHFOR**	7%**
No. of Patches (NUMP) 1 forest (40,50,60,70)	GCNUmP1	7%**

Significant environmental variables and their contribution to the explanation of variance in the species dataset when used alone.

#### Environmental justification to clustering of sites based on species

The best explanatory variables were filtered in a stepwise procedure. A discriminant canonical analysis then used linear combinations of the original environmental variables standardised to predict belonging to the six groups of sites previously defined by indirect clustering and indicator species (Table 3). Four groups out of 6 are well characterized by environmental factors with more than 80% of the sites attributed to the correct group.

**Table 3:** Environmental influence for species clusters

	**Well classified sites**	**Misclassified sites**						**Linear discriminant function for proposed cluster of sites**										
**Indicator species and groups defined using clustering & indval**		**Group 1**	**Group 2**	**Group 3**	**Group 4**	**Group 5**	**Group 6**	**Minimum rain (minrain)**	**Rain warmer quarter (rainwarmq)**	**Temperature range (dayranget)**	**Annual wetness index (men3wian)**	**Range wetness at site (ra3wian)**	**Annual greeness range (men3vira)**	**Dense forest (forest)**	**Mosaic forest shrub (mosaic)**	**Mosaic forest crop (shrub)**	**Number forest patch (patchfor)**	**Shrimp farms (shrimp)**
Group 1 *An. minimus*	33% (3/9 sites)			KMDA, KPLB, KPVA		VBKA, VBKB	VBHB	-1.32	-1.73	-1.93	2.55	-0.60	-1.61	0.80	2.92	0.41	0.91	-0.43
Group 2 *An. peditaeniatus*	85% (11/13 sites)			VTYB	LSYA			1.29	0.83	4.62	4.15	-1.28	0.34	-2.35	-2.58	0.16	0.10	0.20
Group 3 *An. dirus*	91% (10/11 sites)	VDGA						-1.35	-2.46	-1.77	5.52	-1.56	-0.75	1.02	0.47	-1.76	1.85	-0.08
Group 4 *An. vagus*	48% (11/23 sites)	VTHB	VKTA, VQTA, LKMA	VLDA, VSLB, VTYA, KKPB		LBKA, VHGA, VHGB, VLCA		1.16	0.86	0.01	5.09	-0.40	-0.77	-0.75	-0.66	-1.03	0.70	-0.19
Group 5 *An. sinensis*	82% (9/11 sites)				VSLA		VTGB	0.73	1.39	-1.35	0.61	0.72	-0.03	0.06	0.69	0.24	-0.55	-0.25
Group 6 *An. epiroticus*	100% (19/19 sites)							-1.30	-0.17	-0.45	-13.75	2.13	1.92	1.52	0.51	1.83	-2.10	0.48

For each of the six group defined by species/sites indirect clustering, the most indicative species is indicated under the group. The percentage (number of sites) correctly classified by the environmental analysis is provided as well as the number and name of misclassified sites and groups in which they were placed. The coefficients of the linear discriminant function are provided for each environmental factor and each group.

The *An. peditaeniatus* group 2 is reasonably well classified (85%). Numerous species are indicative of this group. A high value of wetness index (mean3wian) and temperature range (DAYRANGET) characterize this group as well as negative correlation with dense forest (FOREST) and forest mosaic (SHRUB). The *An. dirus* group 3 is well characterized (91%) with positive correlation with mean annual wetness vegetation index (men3WIAN), number of forest patch (PATCHFOR) and presence of dense forest (FOREST) and negative correlation with most of the other factors. Group 5 is characterized by *An. sinensis* and correlated with a high value of rainfall in the warm quarter (RAINWARMQ) and low temperature range (DAYRANGET). *An. epiroticus* group 6 show a strong negative correlation with annual wetness index (men3wian) but the expected correlation with percentage of shrimp farm surface (SHRIMP) is quite low while still being higher than for other groups. Quantitative parameter derived from remote sensing vegetation index such as the annual wetness index seem to provide the best tools for discrimination between the well characterized groups.

## Discussion

**Figure 5:**
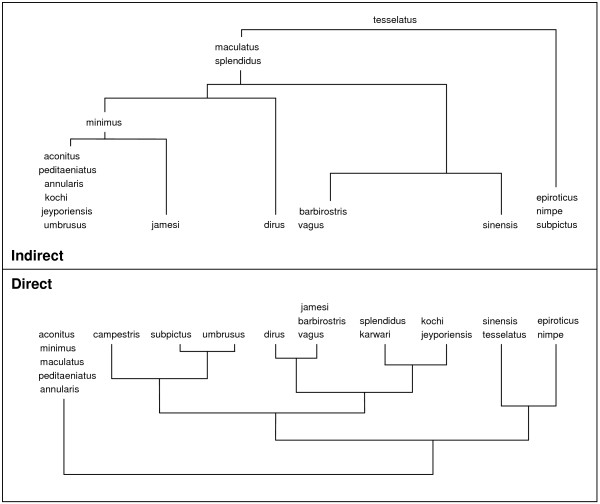
**Comparing direct and indirect species assemblage based on Ward clustering method.** Both methods are based on asymmetrical similarity coefficient. Indirect method is based on ward clustering of sites according to species and analysis of indicative value for each species at each node (see Figure 3). Direct clustering groups species according to log abundance in sites.

Six species assemblages could be defined in this study out of which four could also be significantly characterized by a different environment. Comparison between indirect and direct clustering method, shows that indirect analysis better handled widespread species like *An. tessalatus* and *An. splendidus* (Figure 5). *An. maculatus* is found in 52 sites out of 88 sites and can be considered as a widespread species. It is important however to keep in mind that *An. maculatus* is a complex of sibling species. If the analysis brings some light in the association between species, the main vector *An. dirus s.s.* is unfortunately not associated with any species and presence of another species cannot be used as an indication of potential presence of this elusive species. Unfortunately, *An. minimus* is also not strongly associated to other species and *An. sundaicus* is the most indicative species of a group of sites. This last species can be associated to *An. nimpe* and *An. subpictus*. However the habitat of *An. sundaicus* is already well characterised and there is no need for an additional indicator of presence for this species. The results are thus not optimal for operational use.

The methodology developed here addresses the major issues linked to analysis of adult *Anopheles* species associations. First, abundance is log transformed to smooth the differences in abundances potentially linked to differential response to sampling while avoiding losing too much information. Second, asymmetrical similarity coefficients are used to give less weight to absence and discard double absence [27]. Third, the indirect cluster analysis method IndVal [28] deals elegantly with widespread species, which are generally difficult to identify in direct clustering analysis and generally placed with one or another specialized group or considered as outliers. Indval seems to be a good alternative to the more widely used Twinspan method [42], which compares relative abundance between species and might thus produce misleading results. Here species are evaluated independently from each other. While seldom used in the field of mosquito related research the Indval index is increasingly used in the field of ecology and many tools are available freely online for the user (R project library labdsv). In the field of vegetation sciences, a similar index is used called the phi coefficient of association and derived from the Pearson correlation [43,44]. Recently De Caceres and colleague [45] compared IndVal with the phi coefficient and conclude that the correlation coefficient is more suited to determine species ecological preferences amongst groups of sites but indicator values are the most adapted to determine species assemblages.

Four groups could be discriminated using environmental information, including groups associated with *An. dirus*, *An. pediaeniatus*, *An. sinensis* and *An. epiroticus*. *Anopheles* of the region are very diverse and have different bionomics [46], some of which are directly influenced by the environment. Vegetation indices derived from satellite imagery seem to pick up some of these influences and this might offer the opportunity to work with more accurate information in time. Indeed, preliminary analysis (not shown here) used three detailed national land cover dataset (one per country) but information was not available for the same year or with the same legend for all the countries making regional analysis difficult and the analysis did not show significant results. The GLOBCOVER product [47] with a 300 m resolution used in this analysis seems to be sufficient to bring the necessary information for the analysis and has the advantage of being consistent over the three countries. While potential for regular updates will probably provide up to date information in the future [48], currently, only vegetation indices can give timely information. The greenness indices performed remarkably well in the analysis with the wetness index (NDWI) and the greenness index (NDVI) explaining alone 18% and 16% of the variance (Table 2). Those indices are increasingly available freely and on a regular basis for every square kilometre or even finer scale over the globe. The fact that they can explain a larger part of the variance than land cover based indices is of interest because contrarily to greenness indices, land cover layers are time consuming to produce and integrate errors due to the classification of numerical reflectance values into classes of land cover. The final user has however to keep in mind that quality of those indices might vary according to the source imagery or the processing chain [49]. Using greenness indices might thus be a good option for operational surveillance of environmental changes.

### Seasonal influence and sampling strategies

The sampling design adapted for monitoring purposes is not optimal for exploring biodiversity issues, but is, however, the only type of data available on a wide region as it would be impossible to survey all these villages in the same time*.* This is of importance because the composition of a mosquito population can vary greatly from one week to another according to the rain history. Sites were selected according to two criteria: location in area of malaria transmission and abundance of known or suspected vectors. Indeed, in the dry season, very few mosquitoes are encountered, and in the middle of the rainy season, it is often impossible to reach villages, particularly in forested zones. The assessment of the sampling exhaustivity of the dataset is difficult to estimate in such a wide region and targeting so many species. Indeed the number of species present depends of the type of ecosystem. In some places sampling during three weeks would gather only one species and in other places this would depend of the rain occurrence two weeks before. Surveys were more numerous in Vietnam which have greater technical capabilities, more teams and experience and which routinely carry out field surveys.

## Conclusions

In an attempt to reconcile research and application this article presents a different approach: using the abundant entomological data made available through monitoring programs and available environmental information to extract valuable knowledge for malaria control staff in the field. To compensate for the lack of standardization in the entomological dataset, the study characterized not only few species but species association and their relation to the environment.

The study managed to get around the imperfection inherent to the entomological dataset by using an adapted method based on association and freely available up-to-date products derived from remote sensing techniques. Slight modification in the collection of monitoring data could greatly improve the analysis. The results are limited by how representative the sampling design has been but it is difficult to decide a minimum number of necessary collection days to capture the whole diversity when working with very diverse collection sites [50]. If transversal study could bring the best information on seasonal variation, surveying each site once before and once after the rainy season could help to have a first idea of influence of the season. The indicator value presented in this study could bring information of interest to the entomologist. Building the same methodology on a more standardized dataset collected according to the season could help characterize sites and season associated to vector species and better focus malaria control effort on specific habitat. The results have predictive power only for sites with a habitat similar to those used to find the indicator species [51]. In our case, only suspected malarious areas were surveyed and no information is thus available in other regions of the country. In a region with such a fast-changing environment it would be useful to investigate at least a few sites in each ecological habitat.

Identified indicator species should be further investigated using independent dataset for confirmation of indicator species such as developed in Mc Geoch [52]. While the use of indicator value is here useful to investigate associations between mosquito species, adult mosquitoes such as studied here are probably not the best bio-indicators for particular sites or to predict environmental changes, and this is particularly because of the difficulties linked to sampling and micro-variation in population due to rain history in the previous days.

## Abbreviations

An: *Anopheles*; CCA: Canonical Correspondence Analysis; CRU: Climate Research Unit; CT: Abiotic factors (climatic trends and topography); DAYRANGET: Temperature range; DEM: Digital elevation model; DYNMAP: Dynamic Mapping project; FOREST: Dense forest; GC: Variables derived from land cover GLOBCOVER; GIS: Geographical Information System; INDVAL: Indicator Value method; MALVECASIA: Malaria vector insecticide resistance project SEA; Maxmint: Highest minimal monthly temperature; men3VIRA: Yearly vegetation greenness variation (season); men3WIAN: Mean annual wetness index in 3-km buffer; MERIS: Medium Resolution Imaging Spectrometer; MINRAIN: Minimum rain; MOSAIC: Mosaic vegetation and crop; ND: Vegetation indices; NDVI: Normalized Difference Vegetation Index; NDWI: Normalized Difference Water Index; PATCHFOR: Number of forest patches; PCoA: Principal Coordinates Analysis ordination; ra3WIAN: Range of variation of wetness index in 3-km buffer; RAINWARMQ: Precipitation of the warmest quarter; s.l.: *sensu lato*; s.s.: *sensu stricto*; SEAGIS: Pilot GIS for malaria in SEA; SEASONRAIN: Rainfall pattern; SHRIMP: Shrimp farms; SHRUB: Mosaic shrub land evergreen; SPOT: Systeme Probatoire pour l’Observation de la Terre; UPGMA: Unweighted Pair Group Method Arithmetic Mean; USGS: US Geological Survey; XY: Spatial geographical coordinates.

## Competing interests

The authors declare that they have no competing interests.

## Authors’ contributions

VO conceived the study, performed the statistical analysis and drafted the manuscript, MD made substantial contributions to conception and design of the methodology as well as results interpretation, PD participated in the design of the study and results interpretation, MC participated in the design of the study, analysis and interpretation of the results and supervised the work at all stage. All authors revised, read and approved the final manuscript

## Supplementary Material

Additional file 1 **Technical summary of IndVal method. **Short description and equations for calculation of IndVal indicator value. Click here for file
