# Supplementary material for: Anopheles species associations in Southeast Asia: indicator species and environmental influences
Source: Parasit Vectors. 2013 May 4;6:136. doi: 10.1186/1756-3305-6-136 (PMC3658986; doi:10.1186/1756-3305-6-136)
Supplement: Additional file 1 — Technical summary of IndVal method. Short description and equations for calculation of IndVal indicator value. [file 1756-3305-6-136-S1.doc]

**Additional file 1: Summary of INDVAL technical description**

**IndVal** is a new and simple method to find indicator species and species assemblages characterizing groups of samples. The novelty of the approach lies in the way it combines a species' relative abundance to its relative frequency of occurrence in the various groups of samples. Indicator species are defined as the most characteristic species of each group, found mostly in a single group of the typology and present in the majority of the sites belonging to that group. This duality, which is of ecological interest, is rarely completely exploited in such analyses. In these cases, species occupying only one or two sites in one habitat group and present only in that group (rare species) receive the same indicator value as species occupying all sites of that habitat group and found only in that group. There is an important difference between these two species, however. The first one is an asymmetrical indicator: its presence cannot be predicted in all sites of one habitat, but contributes to the habitat specificity. The second species, on the contrary, is a true symmetrical indicator: its presence contributes to the habitat specificity and one can predict its presence in all sites of the group. To take into account the second part of the symmetric indicator species definition in the expression of the indicator value, the new index was developed. For each species *i* in each site group *j*, we computed the product of A*_ij_*, the mean abundance of species *i* in the sites of group *j* compared to all groups in the study, by B*_ij_*, the relative frequency of occurrence of species *i* in the sites of group *j*, as follows:

**A*_ij_* = Nindividuals*_ij_*/Nindividuals*_i._***

**B*_ij_* = Nsites*_ij_*/Nsites*_.j_***

**IndVal*_ij_* = A*_ij_* * B*_ij_* * 100**

where **IndVal** is the Indicator Value of species *i* in site cluster *j*. In the formula for **A*_ij_***, which is a measure of **specificity**, Nindividuals*_ij_* is the mean number of individuals of species *i* across sites of group *j*, while Nindividuals*_i._* is the sum of the mean numbers of individuals of species *i* over all groups. We use the mean number of individuals in each group, instead of summing the individuals, because this removes any effect of the number of sites in the various site groups, and of differences in abundance among sites belonging to the same group. A*_ij_* is maximum when species *i* is only present in cluster *j*. In the formula for **B*_ij_***, which is a measure of **fidelity**, Nsites*_ij_* is the number of sites in cluster *j* where species *i* is present, while Nsites*_.j_* is the total number of sites in that cluster. B*_ij_* is maximum when species *i* is present in all objects of cluster *j*. Quantities A and B must be combined by multiplication because they represent independent informations about the species distribution. Final multiplication by 100 produces percentages. The indicator value of a species *i* for a typology of sites is the largest value of IndVal*_ij_* observed over all groups *j* of that typology: IndVal*_i_* = max [INDVAL*_ij_*] This index is maximum (= 100%) when all specimens of a species are found in a single group of samples AND when the species occurs in all samples of that group. The basic idea is to measure the species indicator value for all the levels of a hierarchical typology. It is assumed that, the IndVal index changes along the hierarchical typology, being high and decreasing after for generalist species or being low and increasing after for more specialized species when the sample groups are more numerous. The statistical significance of the species indicator values is evaluated using a randomization procedure.
